# Upper airway viruses and bacteria detection in clinical pneumonia in a population with high nasal colonisation do not relate to clinical signs

**DOI:** 10.15172/pneu.2015.6/636

**Published:** 2015-12-01

**Authors:** Anne B. Chang, Heidi Smith-Vaughan, Theo P. Sloots, Patricia C. Valery, David Whiley, Jemima Beissbarth, Paul J. Torzillo

**Affiliations:** 17 Menzies School of Health Research, Charles Darwin University, Casuarina, Australia; 27 Department of Respiratory and Sleep Medicine, Queensland Children’s Health Services and Queensland Children’s Medical Research Institute, Queensland University of Technology, Herston, Australia; 37 School of Medicine, Griffith University, Australia; 47 Sydney Medical School, University of Sydney, Sydney, Australia; 57 Royal Prince Alfred Hospital, Sydney, Australia; 67 Queensland Paediatric Infectious Diseases Laboratory, Sir Albert Sakzewksi Virus Research Centre, Queensland Children’s Health Services, Herston, Australia; 77 Department of Respiratory and Sleep Medicine, Queensland Children’s Hospital and Health Service, South Brisbane, Queensland 4101 Australia

**Keywords:** microbiology, pneumonia, radiology, Aboriginal, child, hospitalised

## Abstract

Indigenous Australian children have high (up to 90%) rates of nasopharyngeal microbial colonisation and of hospitalisation for pneumonia. In Indigenous children hospitalised with pneumonia in Central Australia, we describe the nasopharyngeal detection of viruses and bacteria and assessed whether their presence related to signs of pneumonia (tachypnoea and/or chest in-drawing) on hospital admission and during subsequent days. Nasopharyngeal swabs (NPS) and data were prospectively collected from 145 children (median age = 23.5 months, interquartile range [IQR] 8.7–50) hospitalised with pneumonia at Alice Springs Hospital, Australia, between April 2001 and July 2002. The cohort was enrolled in a randomised controlled study using zinc and/or vitamin A supplementation. NPS were taken within 24 hours of hospitalisation and kept frozen at-80°C until analysed in 2014. Polymerase chain reaction (PCR) was used to detect *Moraxella catarrhalis, Haemophilus influenzae, Streptococcus pneumoniae, Staphylococcus aureus, Chlamydophila pneumoniae, Mycoplasma pneumoniae*, and 16 respiratory viruses. Uni- and multi-variate analyses were used to examine the relationships. One or more organisms were present in 137 (94.5%) NPS; 133 (91.7%) detected ≥ 1 bacterium, 34 (37.2%) for ≥ 1 virus and 50 (34.5%) were positive for both viruses and bacteria. *C. pneumoniae (n* = 3) and *M. pneumoniae (n* = 2) were rare. In multi-variate analyses, age < 12 months (odds ratio [OR] 6.6 [95% confidence interval {CI} 1.7–25.4]) and fever (OR 4.1 [95% CI 1.7–10.4]) were associated with tachypnoea and chest in-drawing. However the presence of bacteria and/or virus type was not associated with tachypnoea and/or chest in-drawing on admission or during recovery. In children with high nasopharyngeal microbial colonisation rates, the utility of NPS in determining the diagnosis of clinical pneumonia or duration of tachypnoea or in-drawing is likely limited. Larger cohort and case-control studies are required to confirm our findings.

## 1. Introduction

Reported hospitalisation rates for acute lower respiratory infections (ALRIs), including pneumonia, in Indigenous Australian children from remote areas are comparable to and, in some regions, higher than those for children in developing countries [1]. Despite the burden, to date, there has been only one study that has examined upper airway viruses and bacteria in Indigenous Australian children from remote communities who have been hospitalised for ALRI [2]. This study was conducted prior to the availability of molecular diagnostic methods [2].

There are few studies that have used the World Health Organization’s (WHO) [3] clinical diagnostic criteria together with current extended molecular methods to examine the relationship between both upper airway viruses and bacteria and clinical and/or radiologically confirmed pneumonia, and its outcomes, in children. The majority of studies have focused on specific viruses [4,5] or bacteria [6,7,8], with few assessing whether upper airway microbiology predicts clinical symptoms. There are none that have been conducted in a population with a similar pneumonia risk to that of Central Australian Indigenous children. Use of nasopharyngeal swabs (NPS) is convenient, as obtaining lower airway specimens in very young children generally requires invasive procedures (e.g. bronchoscopy or lung punctures). However, NPS taken at hospitalisation may not reflect clinical symptoms or outcomes as nasopharyngeal (NP) carriage in this population is high (up to 90% of asymptomatic children) [9,10] and detection of organisms by polymerase chain reaction (PCR) at a single point in time does not necessarily equate to active infection. Further, carriage of NP viruses is as high as 45% of asymptomatic hospitalised children [11].

In 145 Central Australian Indigenous children hospitalised with pneumonia, we describe the point prevalence of NP bacteria and viruses in these children and assessed whether viruses and bacteria detected in the NP related to the WHO clinical definition for pneumonia (tachypnoea and/or chest in-drawing) on admission and the duration of these signs. Given the usually high level of NP carriage of bacteria and viruses in children without pneumonia in our target group, we hypothesised that there was no relationship between these upper airway microbes and clinical symptoms.

## 2. Methods

### 2.1 Setting

The study was undertaken between April 2001 and July 2002 at the Alice Springs Hospital, Northern Territory, Australia, the sole hospital servicing the region. The Alice Springs Hospital is a 189-bed hospital in the Central Australian desert region and serves a population of approximately 60,000 persons dispersed across 1.6 million^2^ kilometres. Forty per cent of the population is Aboriginal, most of who reside in remote communities. At the time of the study, Central Australian Aboriginal children had been receiving *Haemophilus influenzae* type b vaccine (Hib), PRP-OMP conjugate, since 1992 at ages 2, 4 and 12 months. In June 2001 (during study recruitment), the 7-valent pneumococcal conjugate vaccine (PCV7) was introduced to the routine schedule, administered at 2, 4, and 6 months of age with a booster dose of the 23-valent pneumococcal polysaccharide vaccine at 18 months of age. In our setting, children with clinically suspected pneumonia are treated in community clinics according to standard treatment protocols and retrieval to hospital occurs when oxygen is required or the child “fails to respond to treatment”.

### 2.2 Study design

We used data collected prospectively during a randomised controlled trial (RCT) of zinc and vitamin A supplementation in 215 Central Australian Aboriginal children (aged <11 years) hospitalised with pneumonia [12]. All children enrolled in the trial had an illness with cough, tachypnoea, and documented fever (≥38.5°C) or pneumonia diagnosed by chest radiograph (CXR), when referred to the hospital. Blood culture was taken as part of the clinical routine care in the hospital.

The inclusion criteria for the RCT [12] were: (a) an illness with tachypnoea and either documented fever (temperature ≥38.5°C) or chest in-drawing; or (b) pneumonia diagnosed by CXR. Tachypnoea was defined as respiratory rate at or above 60 breaths per minute (age <2 months), 50 breaths per minute (age 2–11 months), or 40 breaths per minute (age 12–59 months), or an arbitrary cut-off of 30 breaths per minute at age 5 years and over. Exclusion criteria for the RCT were presence of bronchiolitis syndrome (wheezing, coryza), or chronic lung, gastrointestinal, or neurological disease.

Informed consent was obtained from the carer(s) by a research nurse or a member of the research team. Detailed clinical data (antibiotics prior to admission, fever, duration of fever, tachypnoea, chest in-drawing) and demographic data (gender, birth weight, age, prior pneumonia hospitalisation) were collected using standardised case report forms. NPS were collected after enrollment and within 24 hours of admission to the paediatric ward. For this study, we assessed the signs that compromise the WHO definition for clinical pneumonia (tachypneoa and/or chest in-drawing). Accessory muscle use (for assessment of chest in-drawing) and the child’s respiratory rate were documented after observing the child for 60 seconds after oxygen had been turned off (if applicable). These assessments were undertaken by trained research nurses twice a day but, given the known variability in children’s respiratory status, only the more severe (higher) rate was recorded on a daily basis. The total hours of fever and tachypnoea and total days of chest in-drawing were calculated.

Of the 215 episodes of hospitalised pneumonia included in the RCT, 57 were excluded from this study as swabs were not available and a further 13 were excluded as the swab was not taken within 24 hours of admission. The final dataset for this paper therefore comprised 145 episodes.

### 2.3 Laboratory

NPS, stored in skim milk tryptone glucose glycerol broth, were kept frozen at −80°C until analysed in 2014. We have previously demonstrated that our stored NPS (at −80°C) are valid for viral and bacterial identification and quantification by culture and PCR [13,14]. Both viral and bacterial detection by PCR were undertaken in accordance to established methods in our laboratories [9,13,14,15].

Virus testing was conducted at the Queensland Paediatric Infectious Diseases (QPiD) Laboratory, Queensland, Australia, for 16 viruses according to previously described PCR methods [9,15]. These viruses included human rhinovirus, adenovirus, respiratory syncytial virus (RSV), influenza virus types A and B, parainfluenza virus types 1–3, human metapneumovirus, human coronaviruses (OC43, 229E, NL63, and HKU1), human bocavirus, and human polyomaviruses KI and WU. QPiD Laboratory also performed PCR testing for *Chlamydophila pneumoniae* and *Mycoplasma pneumoniae*. Quantitative PCR for *Moraxella catarrhalis, H. influenzae, Streptococcus pneumoniae* and *Staphylococcus aureus* was performed at the Menzies School of Health of Research Laboratory, Darwin, Australia, according to previously published methods [16,17].

### 2.4 Ethics statement

The primary study was approved by the Central Australian Ethics Committee and the Queensland Institute of Medical Research (Approval number: P326). Informed written consent was obtained from a parent/carer. This study was conducted under the provisions of the Declaration of Helsinki.

### 2.5 Statistics

Chi-squared tests were used to compare categorical variables (fever present, antibiotics prior to admission, age categories, presence of the various bacteria and viruses). For multi-variate analyses, variables with *p* values of < 0.2 in univariate analysis were used in the model to provide adjusted odds ratios (ORs). All statistical analyses were performed using Stata version 12 (USA).

Given the small number of observations of individual viruses, we classified viruses into two groups [11]: Group A = RSV, influenza virus types A and B, human metapneumovirus, adenovirus and parainfluenza viruses types 1–3; Group B = human rhinovirus, human bocavirus, human polyomaviruses K1 and WU, human coronaviruses OC43, 229E, NL63, and HKU1. The grouping of the viruses is controversial. The primary reason for this classification is based on the high prevalence of Group B viruses in asymptomatic children, whereas detection of Group A viruses is mostly associated with respiratory symptoms [11,18,19,20].

## 3. Results

The children’s median age was 23.5 months (interquartile range [IQR] 8.7–50.0); other data are presented in Table 1. In 138 (95.2%) episodes a blood culture was taken: 11 (8.0%) were culture positive; 1 was positive for Hib, 1 for *S. pneumoniae*, 1 for *S. aureus*, and the remainder were considered contaminants.

**Table 1:** Demographic data of the cohort

Characteristic	Number of patients (%)
Gender	
Male	80 (55.1)
Female	65 (44.8)
Birth weight^a^	
<2500 grams	12 (9.0)
≥2500 grams	122 (91.0)
Gestational age^a^	
<37 weeks	15 (11.3)
≥37 weeks	118 (88.7)
Prior pneumonia hospitalisation	
Yes	62 (45.3)
No	75 (54.7)

^a^some data missing

### 3.1 Point prevalence of bacteria and viruses in the NPS

Most swabs (n =137 [94.5%]) were positive for at least 1 organism (Table 2): there was at least 1 bacterium in 91.7% of the NPS, and at least 1 virus in 37.2%. Human rhinovirus and the polyomaviruses K1 and WU were the most commonly detected viruses, 24.7% and 9.0%, respectively. Organisms infrequently detected were *C. pneumoniae* (*n* = 3 [2.1%]), *M. pneumoniae* (*n* = 2 [1.4%]), influenza virus (either A or B) (*n* = 6 [4.1%]), RSV (*n* = 2 [1.4%]), parainfluenza virus type 3 (*n* = 3 [2.1%]), adenovirus (*n* = 2 [1.4%]), bocavirus (*n* = 2 [1.4%]), and coronavirus NL63 (*n* = 1 [0.7%]). There were no positive detections of *S. aureus*, human metapneumovirus, parainfluenza virus types (1 or 2), or coronaviruses HKU1, OC43, or 229E.

**Table 2:** Univariate analysis of child specific and upper airway microbiological factors associated with clinical symptoms of WHO-defined clinical pneumonia in Central Australian Aboriginal children hospitalised with pneumonia (*n* = 145)

Variable, *n* in total cohort	Tachypnoea			Chest in-drawing			Tachypnoea and chest in-drawing		
	Yes	No	OR (95% CI)	Yes	No	OR (95% CI)	Yes	No	OR (95% CI)
	*n* (%)	*n* (%)		*n* (%)	*n* (%)		*n* (%)	*n* (%)	
Age in months									
0 to <12, *n* = 48	19 (36.5)	29 (31.2)	1.9 (0.7–5.3)	19 (51.3)	29 (26.9)	3.8 (1.1–12.6)	10 (45.5)	38 (30.9)	3.3 (0.7–16.3)
12 to <24, *n* = 25	12 (23.1)	13 (14.0)	2.6 (0.8–8.5)	6 (16.2)	19 (17.6)	1.8 (0.5–7.4)	4 (18.2)	21 (17.1)	2.4 (0.4–14.3)
24 to <60, *n* = 45	14 (26.9)	31 (33.3)	1.3 (0.4–3.7)	8 (21.6)	37 (34.3)	1.2 (0.34.6)	6 (27.3)	39 (31.7)	1.9 (0.4–10.3)
≥60, *n* = 27	7 (13.5)	20 (21.5)	Ref	4 (10.8)	23 (21.3)	Ref	2 (9.1)	25 (20.3)	Ref
Antibiotics prior to admission^a^									
Yes, *n* = 24	5 (10.2)	19 (22.1)	0.4 (0.1–1.2)	5 (14.7)	19 (18.8)	0.7 (0.3–2.2)	2 (10.0)	22 (19.1)	0.5 (0.1–2.2)
No, *n* = 111	44 (89.8)	67 (77.9)	Ref	29 (85.3)	82 (81.2)	Ref	18 (90.0)	93 (80.9)	Ref
Fever ≥38.5°C^a^									
Yes, *n* = 48	21 (41.2)	27 (29.0)	1.7 (0.8–3.5)	17 (47.2)	31 (28.7)	2.2 (1.0–4.8)	11 (52.4)	37 (30.1)	2.56 (1.00–6.54)
No, *n* = 96	30 (58.8)	66 (71.0)	Ref	19 (52.8)	77 (71.3)	Ref	10 (47.6)	86 (69.9)	Ref
*Streptococcus pneumoniae* present in NPS									
Yes *n* = 93	34 (65.4)	59 (63.3)	1.1 (0.5–2.2)	24 (64.9)	69 (63.9)	1.0 (0.5–2.3)	16 (72.7)	77 (62.6)	1.6 (0.6–4.4)
No, *n* = 52	18 (34.6)	34 (36.6)	Ref	13 (35.1)	39 (36.1)	Ref	6 (27.7)	46 (37.4)	Ref
*Haemophilus influenzae* present in NPS									
Yes, *n* = 111	42 (80.8)	69 (74.2)	1.5 (0.6–3.4)	24 (64.9)	87 (80.6)	0.5 (0.2–1.0)	16 (72.7)	95 (77.2)	0.8 (0.3–2.2)
No, *n* = 34	10 (19.2)	24 (25.8)	Ref	13 (35.1)	21 (19.4)	Ref	6 (27.3)	28 (22.8)	Ref
*Moraxella catarrhalis* present in NPS									
Yes, *n* = 102	35 (67.3)	67 (72.0)	0.80 (0.4–1.7)	21 (56.8)	81 (75.0)	0.4 (0.2–0.96)	14 (63.6)	88 (71.5)	0.7 (0.3–1.8)
No, *n* = 43	17 (32.7)	26 (28.0)	Ref	16 (43.3)	27 (25.0)	Ref	8 (36.4)	35 (28.5)	Ref
Any bacteria present in NPS^b^									
Yes, *n* = 133	48 (92.3)	85 (91.4)	1.1 (0.3–3.9)	33 (89.2)	100 (92.6)	0.7 (0.2–3.2)	21 (95.5)	112 (91.1)	2.1 (0.3–16.8)
No, *n* = 12	4 (7.7)	8 (8.6)	Ref	4 (10.8)	8 (7.4)	Ref	1 (4.5)	11 (8.9)	Ref
Group A viruses present in NPS									
Yes, *n* = 12	5 (9.6)	7 (7.5)	1.3 (0.3–5.1)	5 (13.5)	7 (6.5)	2.2 (0.5–8.9)	3 (13.6)	9 (7.3)	2.0 (0.3–9.0)
No, *n* = 133	47 (90.4)	86 (92.5)	Ref	32 (86.5)	101 (93.5)	Ref	19 (86.4)	114 (92.7)	Ref
Group B virus in NPS									
Yes, *n* = 47	16 (30.8)	31 (33.3)	0.9 (0.4–1.8)	11 (29.7)	36 (33.3)	0.9 (0.4–1.9)	7 (31.8)	40 (32.5)	1.0 (0.4–2.6)
No, *n* = 98	36 (69.2)	62 (66.7)	Ref	26 (70.3)	72 (66.7)	Ref	15 (68.2)	83 (67.5)	Ref
Any virus present in NPS^c^									
Yes, *n* = 54	19 (36.5)	35 (37.6)	0.9 (0.5–1.9)	15 (40.5)	39 (36.1)	1.2 (0.6–2.6)	10 (45.5)	44 (35.8)	1.5 (0.6–3.7)
No, *n* = 91	33 (63.5)	58 (62.4)	Ref	22 (59.5)	69 (63.9)	Ref	12 (54.6)	79 (64.2)	Ref
Virus and bacteria co detected									
Yes, *n* = 50	18 (34.6)	32 (34.4)	1.0 (0.5–2.1)	12 (32.4)	38 (35.2)	0.9 (0.4–2.0)	9 (40.9)	41 (33.3)	1.4 (0.6–3.5)
No, *n* = 95	34 (65.4)	61 (65.6)	Ref	25 (67.6)	70 (64.8)	Ref	13 (59.1)	82 (66.7)	Ref

Note: Group A viruses = respiratory syncytial virus, influenza virus types A and B, human metapneumovirus, adenovirus and parainfluenza viruses types 1–3; Group B viruses = viruses commonly detected in asymptomatic children and those with acute lower respiratory infection (human rhinovirus, human bocavirus, human polyomaviruses K1 and WU, human coronaviruses [OC43, 229E, NL63 and HKU1]). Combining data for virus and bacteria in the NPS, most swabs (*n* =137 [94.5%]) were positive for at least 1 organism NPS, nasopharyngeal swabs; Ref, reference for multivariate analyses; OR, odds ratio; CI, confidence interval

^a^Some data missing in these variables

^b^Of these, 29 NPS had 2 bacteria, 70 NPS had 3 bacteria, 3 NPS had 3 bacteria; ORs were not significant (data not shown)

^c^Of these, 10 NPS had ≥2 viruses; ORs were not significant (data not shown)

### 3.2 Relationship between NPS microbial, clinical signs and duration of clinical signs

Table 2 presents the relationship between child characteristics, organism identified, and the presence of tachypnoea and/or chest in-drawing. In both univariate and regressions models (data not shown), there were no covariates significantly associated with tachypnoea. In univariate analyses, age, fever (≥38.5°C) on admission, and being *H. influenzae* and *M. catarrhalis* positive on NPS were associated with chest in-drawing. In adjusted analyses only age <12 months (OR 6.6 [95% confidence interval {CI} 1.7–25.4]) and fever (OR 4.1 [95% CI 1.7–10.4]) remained associated with chest in-drawing. Similarly, age <12 months and fever were associated with tachypnoea and chest in-drawing combined in analyses that adjusted for gender, age, prematurity, comorbidity of gastroenteritis, and fever. Presence of bacteria and/or virus type was not associated with tachypnoea and/or chest in-drawing, in multi-variate analyses. No microbiological associations were found in relation to duration of fever, tachypnoea, or chest in-drawing (Table 3).

**Table 3:** Relationship between nasopharyngeal microbiology and duration of symptoms in Central Australian Aboriginal children hospitalised with pneumonia

Nasopharyngeal microbiology	Fever		Tachypnoea		Chest in-drawing	
	Median no. hours (IQR)	*p*-value	Median no. hours (IQR)	*p*-value	Median no. days (95% CI)	*p*-value
*Streptococcus pneumoniae* positive						
Yes	11 (5–15)	0.923	9 (0–19)	0.431	1.76 (1.28–2.23)	0.974
No	9.5 (6.5–24.5)		10 (0–25)		1.75 (1.20–2.30)	
*Haemophilus influenzae* positive						
Yes	11 (5–24)	0.71	8.5 (0–24)	0.661	1.70 (1.29–2.13)	0.641
No	12 (8–24)		10 (10–17)		1.89 (1.18–2.60)	
*Moraxella catarrhalis* positive						
Yes	11 (5–24)	0.763	10 (0–24)	0.788	1.79 (1.27–2.31)	0.829
No	11 (5–27)		10 (0–17)		1.71 (1.24–2.19)	
Group A virus present						
Yes	10 (7–25)	0.966	0 (0–24)	0.739	2.00 (0.48–3.52)	0.552
No	11 (5–24)		10 (0–21)		1.71 (1.35–2.08)	
Group B virus present						
Yes	9 (7–12)	0.219	8 (0–10)	0.128	1.64 (1.02–2.26)	0.619
No	11.5 (5–25)		11.5 (0–24)		1.82 (1.37–2.26)	
Virus and bacteria positive						
Yes	8 (11–15)	0.866	8 (0–12)	0.497	1.67 (1.10–2.23)	0.691
No	11 (5–24.5)		10.5 (0–24)		1.80 (1.34–2.28)	

Note: Group A viruses = respiratory syncytial virus, influenza virus types A and B, human metapneumovirus, adenovirus and parainfluenza viruses types 1–3; Group B viruses = viruses commonly detected in asymptomatic children and those with acute lower respiratory infection (human rhinovirus, human bocavirus, human polyomaviruses K1 and WU, human coronaviruses OC43, 229E, NL63 and HKU1).

CI, confidence interval; IQR, interquartile range

## 4. Discussion

In 145 Central Australian Indigenous children hospitalised with pneumonia, a very high prevalence of bacteria (91.7%) or virus (37.2%) were detected in the NPS and multiple co-infection was common. Mycoplasma (1.4%) and chlamydia (2.1%) were rare. We found no significant relationship between the NP presence of types of bacteria and/or viruses with signs on admission (chest in-drawing, tachypnoea, fever) or the duration of signs.

We are unaware of any published data (PubMed [https://doi.org/www.ncbi.nlm.nih.gov/pubmed] search February 2015) evaluating the relationship between signs of pneumonia and recovery of children hospitalised with ALRI and upper airway bacteria and viruses in a setting with a high acute [1] and chronic [21] respiratory burden. In other settings, there are many studies that have described the positive association between NP microbiology and ALRI symptoms [22] or pneumonia [8,23,24], but few have evaluated signs of WHO-defined clinical pneumonia to both viral and bacteria detection in the upper airways. Some studies assume that what is found in the upper airways is the aetiological cause of the pneumonia [23,24], and others advocated that microbiology (e.g. pneumococcus) identified on NPS can be used to diagnose aetiology of pneumonia [6]. However, we have previously shown that the yield of respiratory virus detection in NP aspirates versus broncho-alveolar lavage is dependent on virus type [25]. Indeed, delineating the aetiological agent of pneumonia is not straightforward when the direct lower airway specimens (e.g. sputum, lung biopsy) are unavailable and in the absence of a positive blood culture [26]. Complexities include the case definition, use of and interpretation of CXR, blood white cell counts and inflammatory markers, depth of investigations, facility type, and patient characteristics [26], as well as the limited specificity of the WHO diagnosis of pneumonia and CXRs [27]. None of the studies have examined recovery of these signs of clinical pneumonia and it is also likely that these studies have limited applicability in settings with a high infectious burden from a young age; e.g. in Indigenous Australian remote communities and very low-income countries. Our study thus contributes to the medical literature on childhood pneumonia, in spite of the limitations of our study as discussed below.

Our negative findings are likely to be influenced by the very high carriage rates of bacteria in this population from an early age [10, 13], resulting in only a very small number of children who were not positive for any organism. The very high carriage rates of the key respiratory bacteria in Indigenous Australian children in remote communities are well established, with respiratory bacteria NP colonisation rate of up to 90% [10]. Also, colonisation occurs very early in life (as young as 2 weeks) [10,28]. From birth, the nasopharynx of Indigenous Australian infants are “colonized with multiple species of respiratory bacteria (M. *catarrhalis, H. influenzae, S. pneumoniae*) at a rate of 5% per day” [28]. Similarly, *M. catarrhalis* (96%), *H. influenzae* (91%), *S. pneumoniae* (89%), and respiratory viruses (59%) were identified in NPS of 114 Indigenous children from remote communities, with and without acute otitis media [13] and without pneumonia.

In contrast to our findings, a German study (a low baseline carriage setting) involving 311 children with ALRI [6] described that bacteria in the NP were associated with some clinical features and outcomes. Among other findings, the study described that children with *S. aureus* positive co-colonisation received more antibiotics (*p* = 0.004), received more frequent CXRs (*p* = 0.05), and stayed longer in hospital (*p* = 0.0003) than those with *S. aureus* single colonisation [6]. However, the study did not examine for viruses and only 102 children had pneumonia (the remainder had a wheezing illness and hence the illness was most likely a viral ALRI). Thus, the German study is incomparable to our study. Our study had low prevalence of RSV and influenza virus, which is not unexpected given that children with bronchiolitis symptoms (e.g. wheezing) were excluded.

Our study was an opportunistic evaluation of NP microbiology within a RCT, and hence the association component of our findings has many limitations. Firstly, the sample size is relatively small and thus we had limited power to detect small differences between groups with certainty, and examination of interactions could not be validly performed. Secondly, we did not have a control group to contemporaneously evaluate if NP carriage in children hospitalised with pneumonia was higher than those hospitalised for other reasons; however, our study’s aim was to evaluate NP microbiology with symptoms and signs of pneumonia and not between groups of children with different diseases. Thirdly, the majority of children (73%) were from remote communities and their clinical presentation at time of admission to the ward would have been influenced by treatment (e.g. antipyretics, oxygen therapy, prior antibiotics) prior to retrieval to hospital. It is likely that more children would have had clinical signs suggestive of pneumonia when they presented to the remote community clinic. However, this scenario of prior treatment and delay prior to management in a major hospital is similar to some regions in developing countries where treatment is initiated in the local clinic and the child transferred to a major hospital if required. Nevertheless, studies conducted at the community level prior to transfer to hospital are needed in this high-risk group to adequately assess predictors of symptoms and signs.

In summary, our study suggests that NPS collected at the time of hospitalisation are likely to be of limited use in determining the diagnosis of pneumonia based on key WHO signs (tachypnoea and chest in-drawing), and for the duration of these signs in children with high NP microbial colonisation rates. Larger cohort and case-control studies are needed to confirm our findings.
